# Supplementary material for: Commercial integrated crop-livestock systems achieve comparable crop yields to specialized production systems: A meta-analysis
Source: PLoS One. 2020 May 7;15(5):e0231840. doi: 10.1371/journal.pone.0231840 (PMC7205283; doi:10.1371/journal.pone.0231840)
Supplement: S1 Fig — Black points represent the mean effect size of each study included in the meta-analysis. White points are hypothetical missing studies infilled by an algorithm to achieve symmetry in the funnel plot. (DOCX) [file pone.0231840.s002.docx]

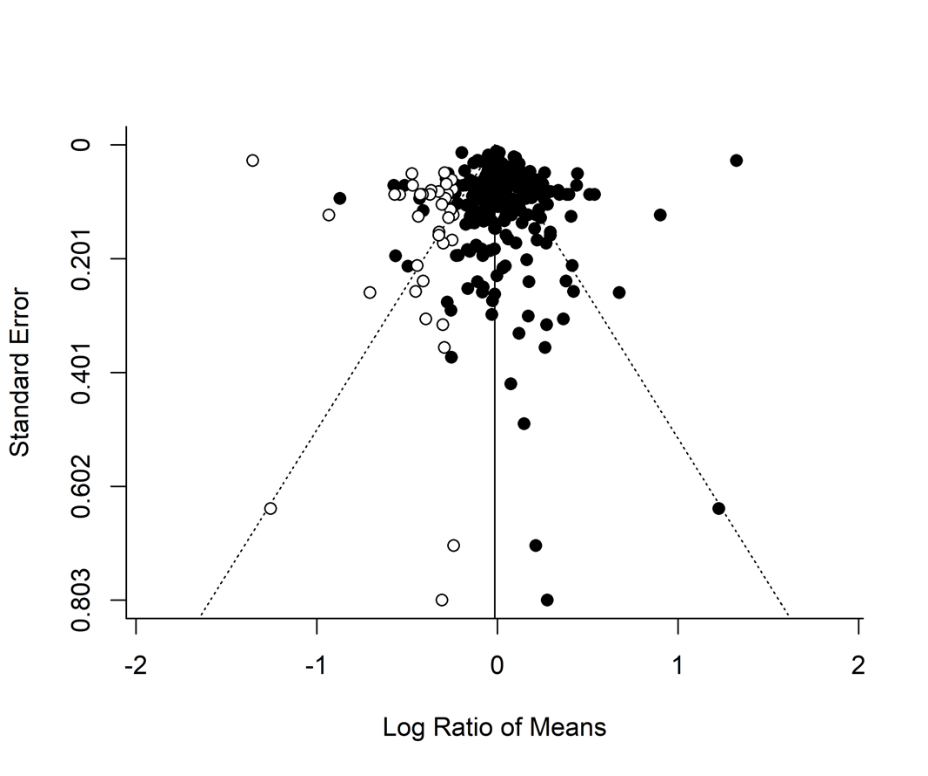


**Suppl Fig S1.** Funnel plot illustrating the trim and fill method used to detect potential plot asymmetry indicating publication bias (Koricheva et al., 2013). Black points represent the mean effect size of each study included in the meta-analysis. White points are hypothetical missing studies infilled by an algorithm to achieve symmetry in the funnel plot.
