# Supplementary material for: Commercial integrated crop-livestock systems achieve comparable crop yields to specialized production systems: A meta-analysis
Source: PLoS One. 2020 May 7;15(5):e0231840. doi: 10.1371/journal.pone.0231840 (PMC7205283; doi:10.1371/journal.pone.0231840)
Supplement: S3 Fig — Effect of ICLS on crop yield relative to unintegrated systems within subgroups for a) climate and b) length of study. Includes observations from dual-purpose cropping systems. Climate categories are derived from the Köppen classification system. Number of observations/number of studies for each category appears in parentheses. Categories with less than 15 observations were omitted from the subgroup analysis. Points represent grazed system yield effect, while the dotted vertical line represents ungrazed system yields. Error bars represent 95% bias-corrected-accelerated bootstrap confidence intervals. Asterisks (*) represent a significant yield response in grazed systems relative to ungrazed systems at the 95% confidence level. (DOCX) [file pone.0231840.s004.docx]

**
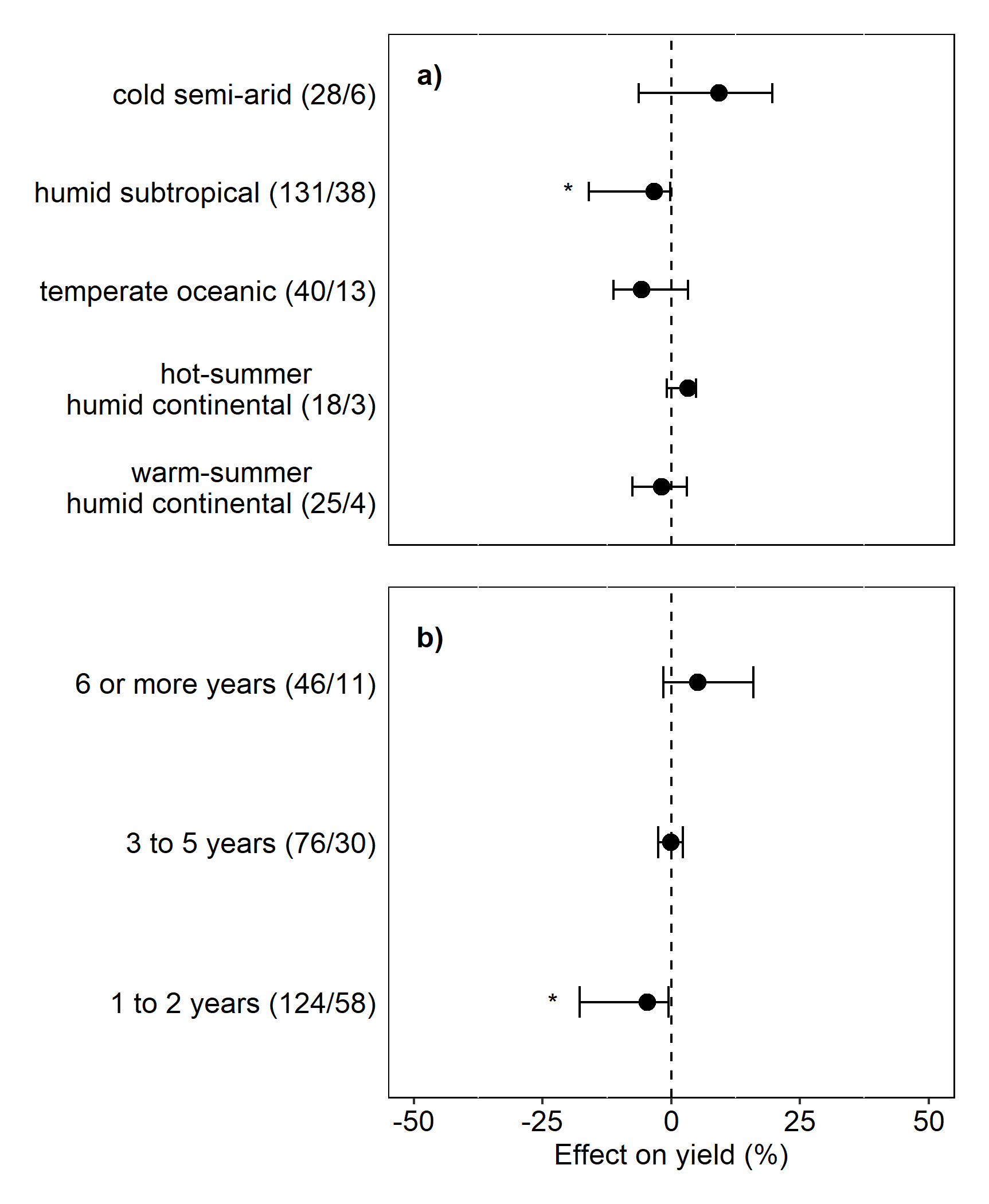
**

**Suppl Fig S3.** Effect of ICLS (including dual-purpose cropping systems) on crop yield relative to unintegrated systems within subgroups for a) climate and b) length of study. Climate categories are derived from the Köppen classification system. Number of observations/number of studies for each category appears in parentheses. Categories with less than 15 observations were omitted from the subgroup analysis. Points represent grazed system yield effect, while the dotted vertical lines represent ungrazed system yields. Error bars represent 95% bias-corrected-accelerated bootstrap confidence intervals. Asterisks (*) represent a significant yield response in grazed systems relative to ungrazed systems at the 95% confidence level.
