# Supplementary material for: Commercial integrated crop-livestock systems achieve comparable crop yields to specialized production systems: A meta-analysis
Source: PLoS One. 2020 May 7;15(5):e0231840. doi: 10.1371/journal.pone.0231840 (PMC7205283; doi:10.1371/journal.pone.0231840)
Supplement: S4 Fig — Effect of ICLS on crop yield relative to unintegrated systems within subgroups for a) livestock type with dual-purpose crop studies excluded and b) livestock type with dual-purpose crop studies included. Number of observations/number of studies for each category appears in parentheses. Categories with less than 15 observations were omitted from the subgroup analysis. Points represent grazed system yield effect, while the dotted vertical line represents ungrazed system yields. Error bars represent 95% bias-corrected-accelerated bootstrap confidence intervals. Asterisks (*) represent a significant yield response in grazed systems relative to ungrazed systems at the 95% confidence level. (DOCX) [file pone.0231840.s005.docx]

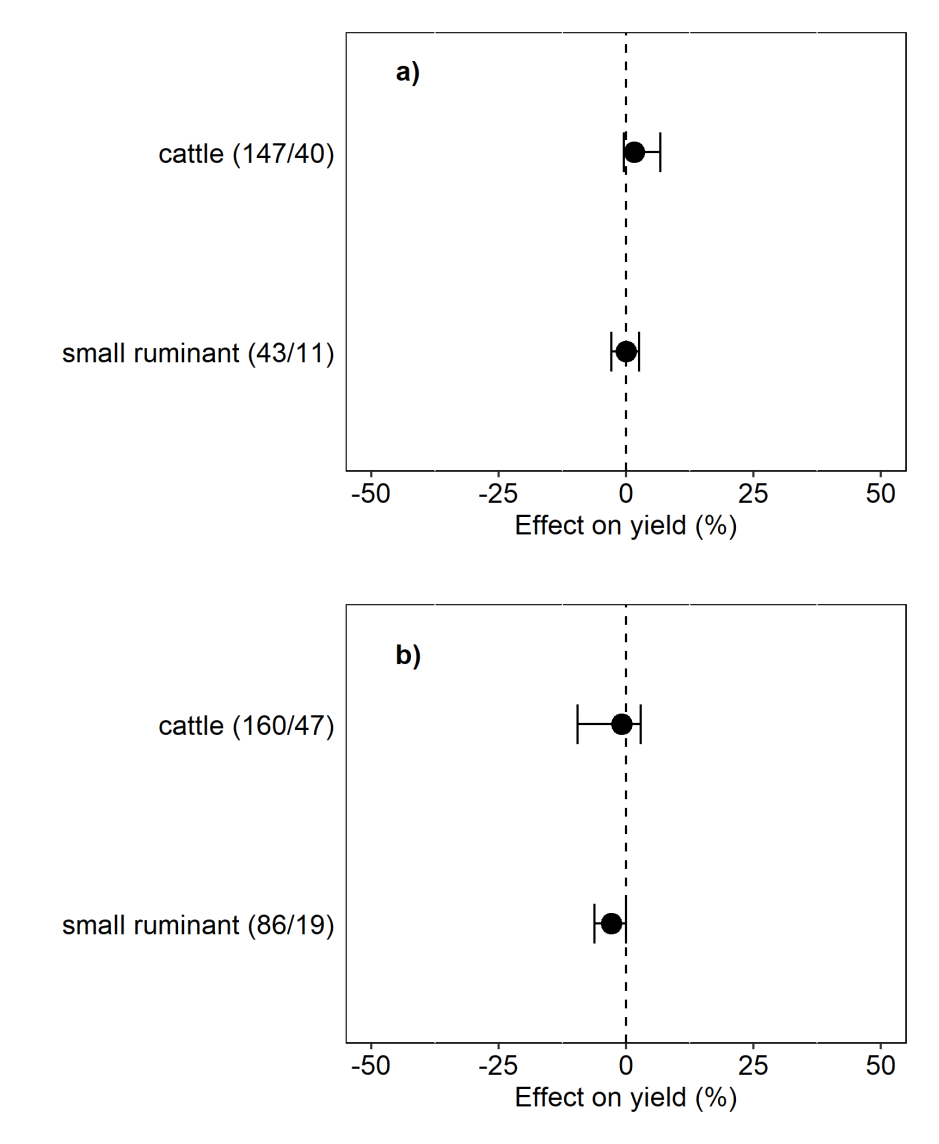


**Suppl. Fig S4.** Effect of ICLS on crop yield relative to unintegrated systems within subgroups for a) livestock type with dual-purpose crop studies excluded and b) livestock type with dual-purpose crop studies included. Number of observations/number of studies for each category appears in parentheses. Categories with less than 15 observations were omitted from the subgroup analysis. Points represent grazed system yield effect, while the dotted vertical line represents ungrazed system yields. Error bars represent 95% bias-corrected-accelerated bootstrap confidence intervals. Asterisks (*) represent a significant yield response in grazed systems relative to ungrazed systems at the 95% confidence level.
